# Supplementary material for: Ammonium Fluoride as Suitable Additive for HILIC-Based LC-HRMS Metabolomics
Source: Metabolites. 2019 Nov 27;9(12):292. doi: 10.3390/metabo9120292 (PMC6950006; doi:10.3390/metabo9120292)
Supplement: Supplementary file 1 [file metabolites-09-00292-s001.zip › supplementary figure 1-5.pdf]

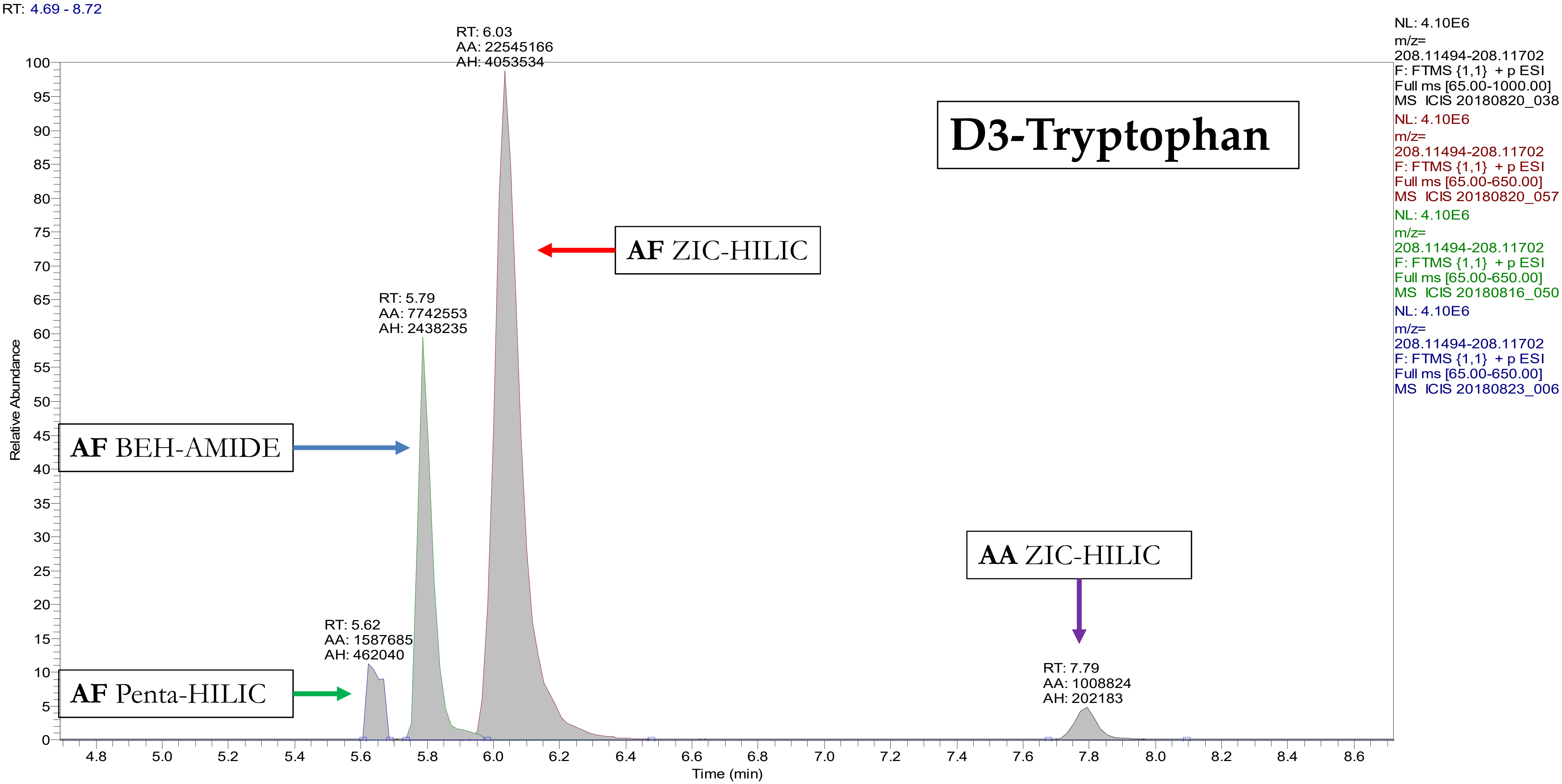

Figure S1. A comparison of the peaks of the deuterated tryptophan between the AA method on the Zic-Hilic clumn, and the AF method performed on 3 different columns: Zic-Hilic, Penta-Hilic and the BEH-Amide columns.

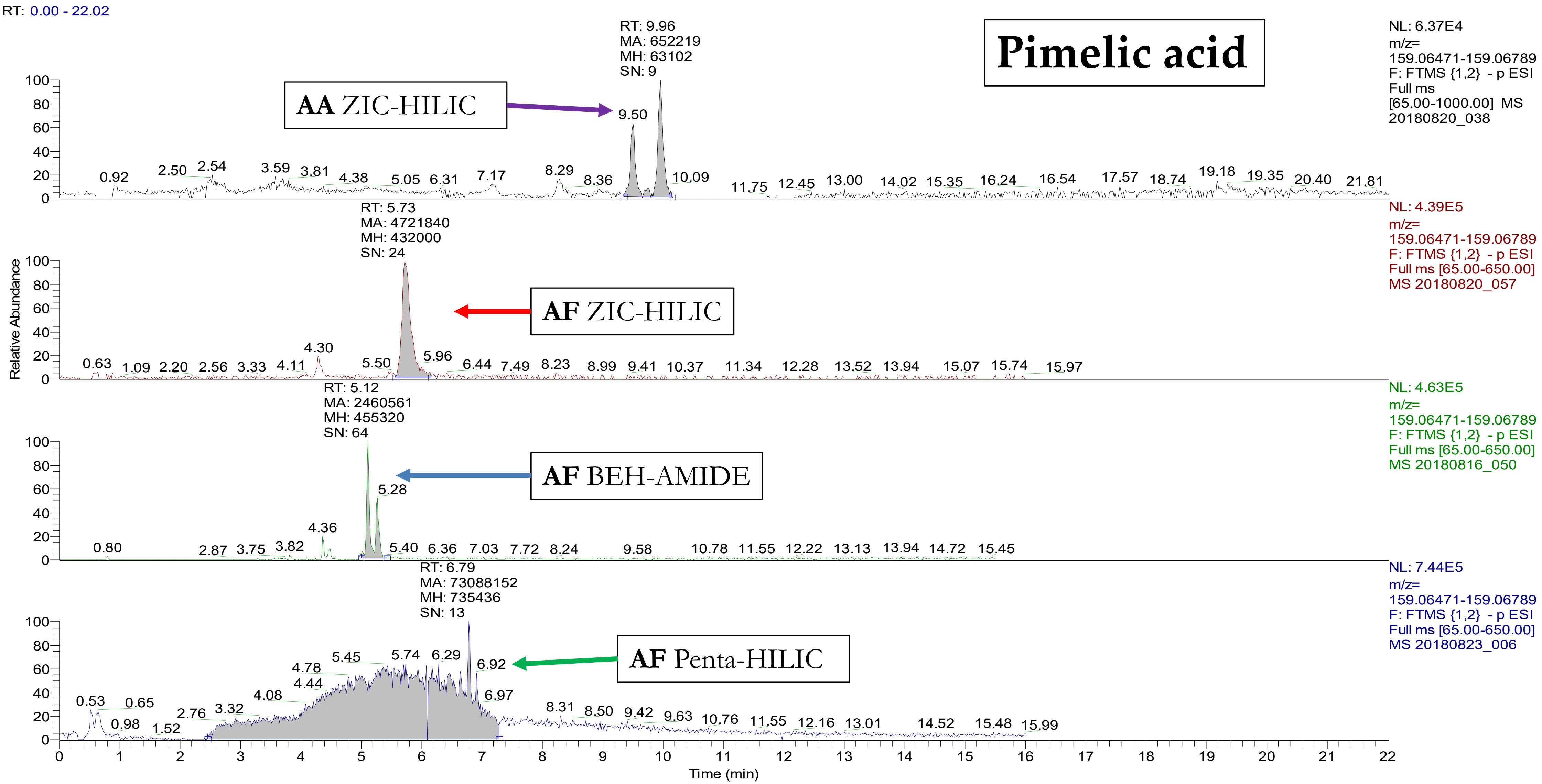

Figure S2. A comparison of the peaks of Pimelic acid between the AA method on the Zic-Hilic column and the AF method on the Zic-Hilic, the BEH-Amide and the Penta-Hilic column.

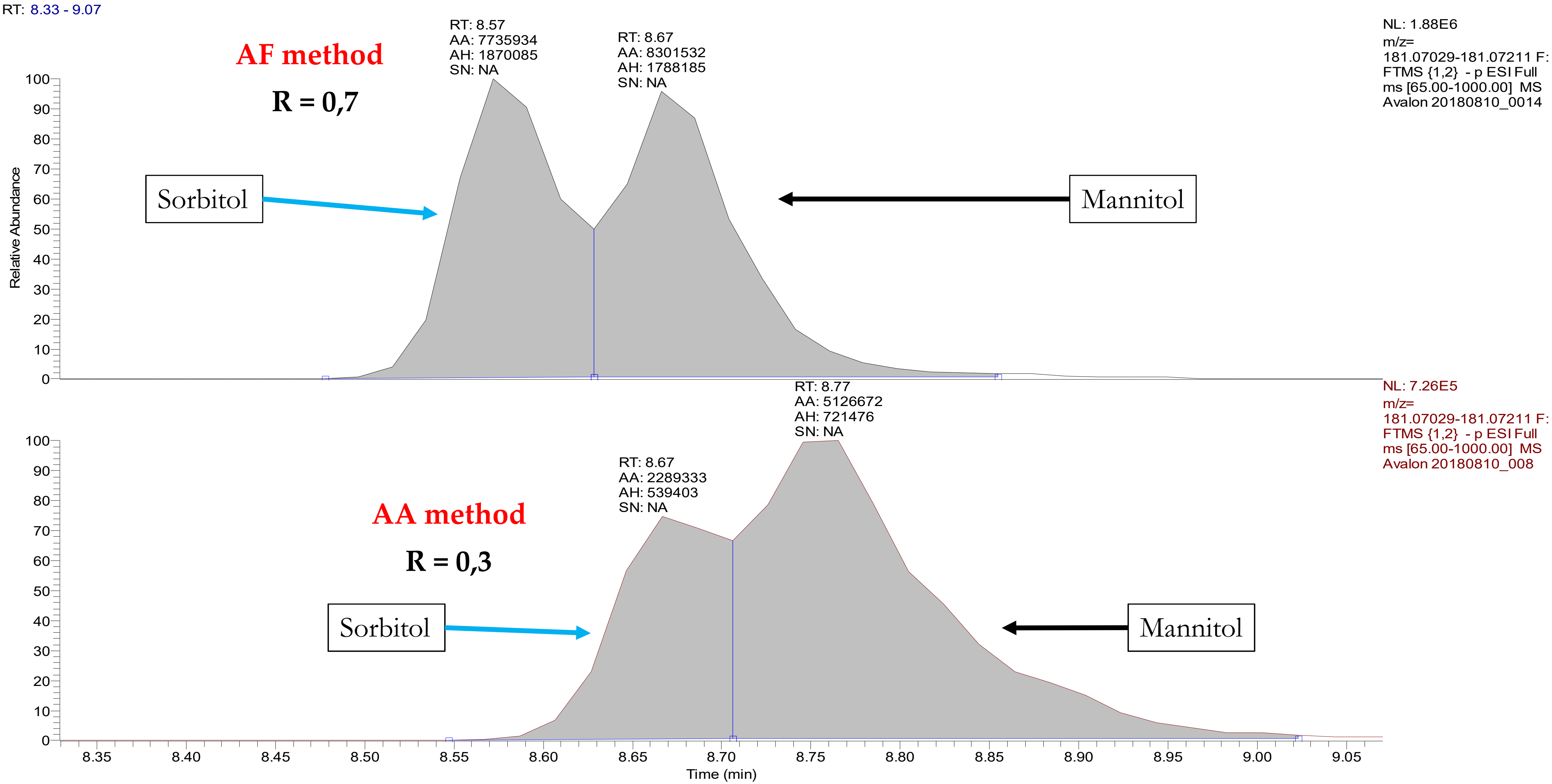

Figure S3. A comparison of the chromatographic resolution of the AA and AF methods for sugars.

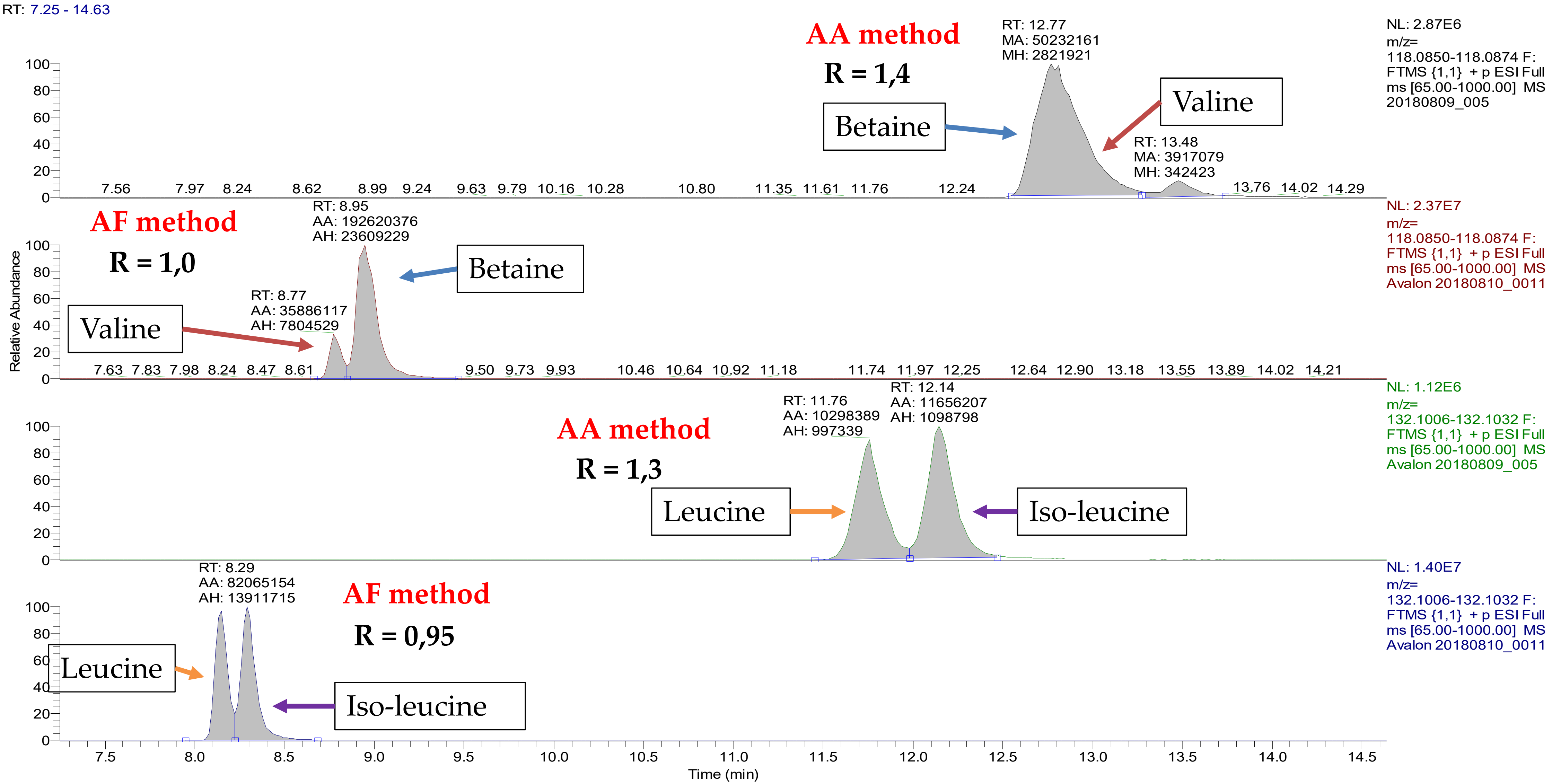

Figure S4. A comparison of the chromatographic resolution of the AA and AF methods for amino-acids.

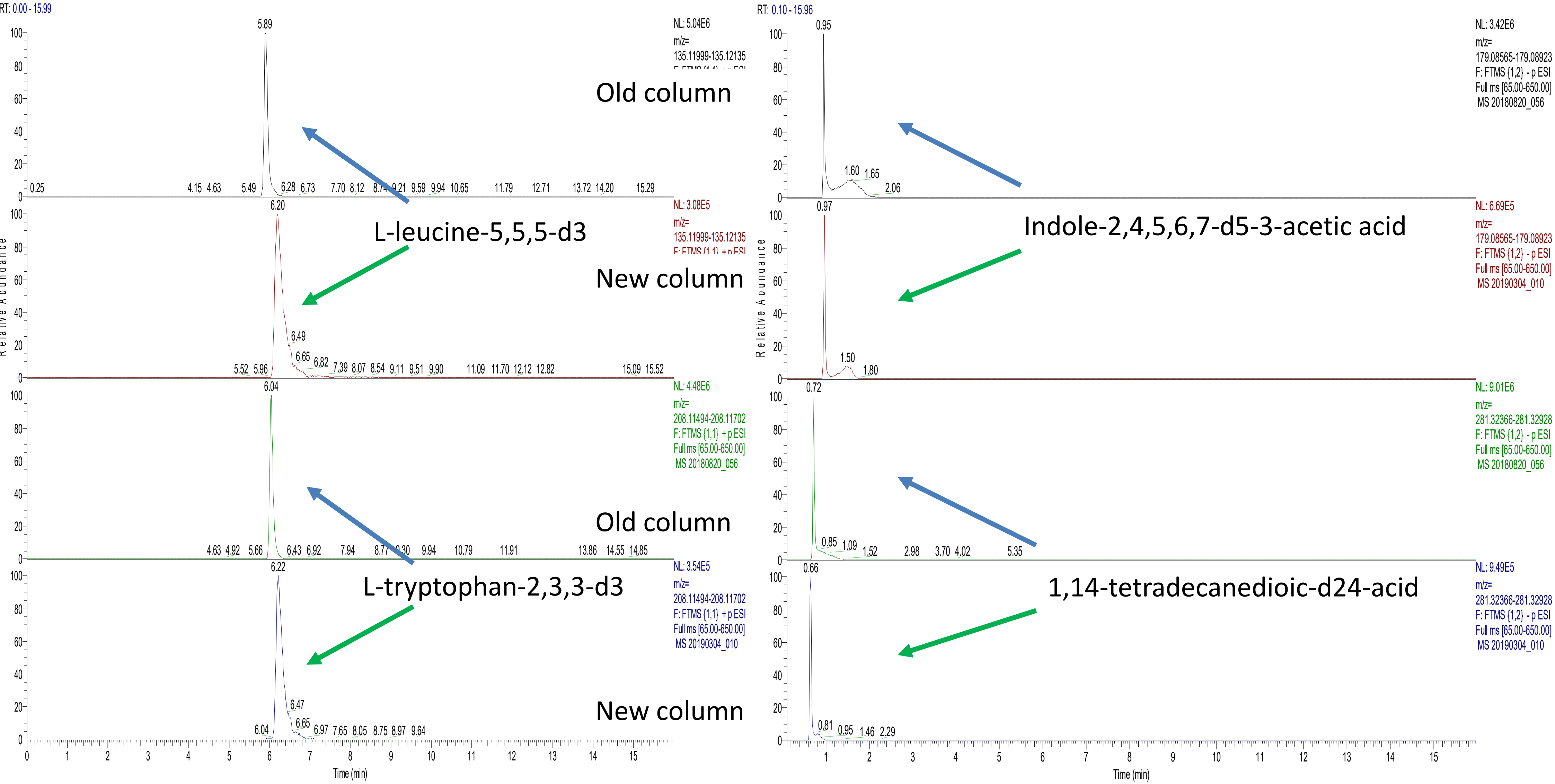

Figure S5. A comparison of the RT shifts between old and new ZIC-Hilic columns both used with the AF method.
